# Streptococcal infection in childhood Henoch-Schönlein purpura: a 5-year retrospective study from a single tertiary medical center in China, 2015–2019

**DOI:** 10.1186/s12969-021-00569-3

**Published:** 2021-06-02

**Authors:** Guo Zhen Fan, Rui Xue Li, Qi Jiang, Man Man Niu, Zhen Qiu, Wei Xia Chen, Hui Hui Liu, Jin Wei Ruan, Peng Hu

**Affiliations:** 1 Department of Pediatrics, Chaohu Hospital of Anhui Medical University, No.64 Chaohu North Road, Hefei, 230022 People’s Republic of China; 2 Department of Pediatrics, The First Affiliated Hospital of Anhui Medical University, No. 218 Ji-Xi Road, Hefei, 230022 People’s Republic of China

**Keywords:** Arthritis, Henoch-Schönlein purpura, Immunoglobulin a, Renal pathology, Streptococcus

## Abstract

**Background:**

The present study focuses on the associations of streptococcal infection with the clinical phenotypes, relapse/recurrence and renal involvement in Henoch-Schönlein purpura (HSP) children.

**Methods:**

Two thousand seventy-four Chinese children with HSP were recruited from January 2015 to December 2019. Patients’ histories associated with HSP onset were obtained by interviews and questionnaires. Laboratory data of urine tests, blood sample and infectious agents were collected. Renal biopsy was performed by the percutaneous technique.

**Results:**

(1) Streptococcal infection was identified in 393 (18.9%) HSP patients, and served as the most frequent infectious trigger. (2) Among the 393 cases with streptococcal infection, 43.0% of them had arthritis/arthralgia, 32.1% had abdominal pain and 29.3% had renal involvement. (3) 26.1% of HSP patients relapsed or recurred more than 1 time within a 5-year observational period, and the relapse/recurrence rate in streptococcal infectious group was subjected to a 0.4-fold decrease as compared with the non-infectious group. (4) No significant differences in renal pathological damage were identified among the streptococcal infectious group, the other infectious group and the non-infectious group.

**Conclusions:**

Streptococcal infection is the most frequent trigger for childhood HSP and does not aggravate renal pathological damage; the possible elimination of streptococcal infection helps relieve the relapse/recurrence of HSP.

## Background

Henoch-Schönlein purpura (HSP), also named immunoglobulin A vasculitis (IgAV), accounts for almost 58.0% of pediatric primary vasculitis and exhibits non-thrombocytopenic purpura with several main features, including abdominal pain, histopathology (leukocytoclastic vasculitis with predominant IgA deposits on skin biopsy), arthritis or arthralgia, renal involvement [1–3]. Based on the European League Against Rheumatism (EULAR) criteria, HSP is defined by purpura or petechiae (mandatory) with lower limb predominance and at least one of main features simultaneously [4]. According to a latest epidemiological study in Taiwan, China, the annual incidence rate of HSP was 9.6 per 100,000 children with the peak age < 6 years old [5]. Generally speaking, HSP is a self-limited condition that lasts an average of 4 weeks, whereas 40–50% of patients undergo renal involvement and aggravate the long-term prognosis of HSP [6]. HSP nephritis (HSPN) develops into end stage in up to 5.1% of patients [7], and is associated with several risk factors, such as persistent purpura (OR = 4.0), severe bowel angina (OR = 3.4) and elevated antistreptolysin O (ASO, OR = 2.2) [8]. The pathogenesis of HSP is still unclear, whereas it may be ascribed to genetic and infectious factors. A retrospective study encompassing 349 Spanish HSP patients and 335 sex ethnically matched controls by López-Mejías et al. [9] identified that HLA-B*41:02 was subjected to a 5.5-fold increase in HSP patients as compared with controls and was related to the susceptibility to HSP (OR = 5.8). Based on the data from 11 studies containing 783 HSP patients, Xiong et al. [10] indicated that helicobacter pylori (HP) was detected in 48.2% of patients, which may promote the elevations of serum IgA, C3 and cryoglobulins.

Numerous epidemiological surveys have noted infection is the most prevalent trigger of HSP. Our recent study analyzed the clinical data of 1200 HSP patients since 2015 to 2017, and showed that potential infections occurred in 611 cases (50.9%), in which streptococcus was the most common infectious agent (17.1%) [11]. In addition, the associations of several rare infection agents (such as Bartonella henselae) with HSP were also reported in previous studies [12]. Streptococcus, Gram-positive facultative anaerobic bacteria, is a common colonizer of the upper respiratory tract and skin of humans [13]. Besides acute infectious inflammation, streptococcus has been proved to be involved in the pathogenesis of some systemic inflammatory diseases, and among them, rheumatic fever is a representative one. In rheumatic fever, streptococcal antigens activate humoral and cell-mediated immune pathways leading to the production of antibodies against streptococcal components, which cross-react with human proteins [14]. Similar mechanisms may also work in HSPN onset. Jauhola et al. [15] retrospectively reviewed 223 pediatric patients with HSP, and found that 40 patients of them had nephritis and streptococcal infection simultaneously. More persuasively, Schmitt et al. [16] performed skin and renal biopsies from 17 HSP patients, and revealed that the deposits of IgA-binding streptococcal M proteins were detected in 80% of skin and 54% of kidneys biopsies respectively.

During the period of 2012–2014, an epidemiological survey by Wu et al. [17] suggested that an average of 29,804.6 patients with streptococcal pharyngitis per 100,000 person-years occurred among Chinese children aged 0–14 years. However, few studies have focused on the associations of streptococcal infection with the clinical phenotypes, relapse/recurrence and renal involvement in HSP children in detail. In this context, the objective of the present study is mainly to fill this gap in Anhui province, China.

## Methods

### Patient selection

The present retrospective study included a total of 2074 children with HSP between January 2015 and December 2019 in Department of Pediatrics, the First Affiliated Hospital of Anhui Medical University. The diagnosis was dependent on EULAR criteria for HSP classification [4]. All cases developed purpura that consisted of the characteristic skin lesions 2–10 mm in diameter, simultaneously accompanied with at least one of the 4 following clinical manifestations: (1) abdominal pain: diffuse abdominal colicky pain with acute onset assessed by history and physical examination, also including intussusception and gastrointestinal bleeding. (2) Histopathology: typically leucocytoclastic vasculitis with predominant IgA deposit or proliferative glomerulonephritis with predominant IgA deposit. (3) Arthritis or arthralgias: arthritis of acute onset defined as joint swelling or joint pain with limitation on motion. Arthralgia of acute onset defined as joint pain without joint swelling or limitation on motion. (4) Renal involvement: proteinuria: > 0.3 g/24 h or > 30 mmol/mg of urine albumin/creatinine ratio on a spot morning sample; hematuria or red blood cell casts: > 5 red blood cells/high power field or red blood cells casts in the urinary sediment or ≥ 2+ on dipstick. The exclusion criteria were: (1) patients with incomplete information; (2) patients unable to comply with the treatment and (3) patients with severe heart, liver, lung, kidney or other organ system diseases. According to the report of Reamy et al. [18], treatment principles for HSP were adopted in the present study. Anti-infectious agents were recommended for those who suffered from an infection. Relapse/recurrence was defined when a patient previously diagnosed with HSP and asymptomatic for at least 2 weeks after treatment, presented again a new flare of cutaneous lesions or other systemic manifestations of the vasculitis [19].

### Data collection

Approval for this research was acquired from the Medical Ethic Committee of the First Affiliated Hospital of Anhui Medical University, and the informed consent was obtained from all parents. Laboratory data included urine tests, fecal occult blood, HP, mycoplasma antibodies (MP-Ab), tubercle bacillus antibody (TB-Ab), TORCH (toxoplasma gondii, others, rubella virus, cytomegalo virus, herpes virus), Epstein-Barr virus (EBV), respiratory pathogens (*Legionella pneumophila*, chlamydia pneumoniae, adenovirus, respiratory syncytial virus, influenza A virus, influenza B virus, rickettsia, parainfluenza virus), creatine kinase (CK), CK-MB, aspartate aminotransferase (AST) and alanine aminotransferase (ALT). Patients’ histories associated with HSP onset (foods, drugs, vaccinations, insect bites) were obtained by interviews and questionnaires.

### Streptococcal serology

An acute serum sample for streptococcal serology was collected during the hospital stay, and a convalescent serum was collected approximately 1 week after the cessation of antimicrobial therapy [20, 21]. ASO titer was measured using nephelometry (Siemens Healthcare Diagnostics, Marburg, Germany). For upper limits of normal, previously published values for children from Chinese mainland of 200 for ASO were used [22]. Seropositivity was defined as (1) a 0.2 log10 rise in titer and a titer ≥200 IU/mL in the convalescent serum or (2) titers of both acute and convalescent sera ≥200 IU/mL.

### Renal pathology

The indications for renal biopsy are as follows: (1) nephrotic syndrome, acute, or chronic renal disease; (2) nephrotic range proteinuria (urine protein/creatinine ratio, > 250 mg/mmol) at 4–6 weeks (if not improving spontaneously); (3) persistent proteinuria—urine protein/creatinine ratio > 100 mg/mmol with > 3 months [23]. Contraindications to percutaneous renal biopsy have included solitary kidney, uncontrollable bleeding diathesis, uncontrolled severe hypertension and inability to cooperate with biopsy. In all cases, the procedure was performed after providing information to parents and obtaining informed consent. Renal biopsy was performed by the percutaneous technique using a Tru-Cut needle (Baxter, Deerfield, IL) under ultrasound control. Renal biopsy specimens were graded based on the established International Study for Kidneys Disease in Children (ISKDC) classification: I, minimal glomerular alterations; II, mesangial proliferation only; III, focal (IIIa) or diffuse (IIIb) proliferation or sclerosis with < 50% crescents; IV, focal (IVa) or diffuse (IVb) mesangial proliferation or sclerosis with 50–75% crescents; V, focal (Va) or diffuse (Vb) mesangial proliferation or sclerosis with > 75% crescents; VI, membranoproliferative-like lesions [24]. The specimens were stained with hematoxylin and eosin, periodic Acid-schiff, and Schiff-methenamine silver and examined by light microscopy. The following proteins were assessed by immunofluorescence: complement (C) 3, IgA, IgM, IgG, kappa and lambda.

### Statistical analysis

Statistical analyses were performed using the statistical package for social studies SPSS V.22.0. Normally distributed continuous data were presented as mean ± standard deviation. Comparisons of the frequencies among groups were analyzed using Chi-square tests. Comparison of mean values between groups was carried out using the independent sample t-test. Comparison of mean values among groups was carried out using one-way ANOVA, and post hoc analysis was calculated using the Student-Newman-Keuls test. All *p* values were two-sided and *p* < 0.05 were considered statistically significant.

## Results

### Demographic features

**Fig. 1:**
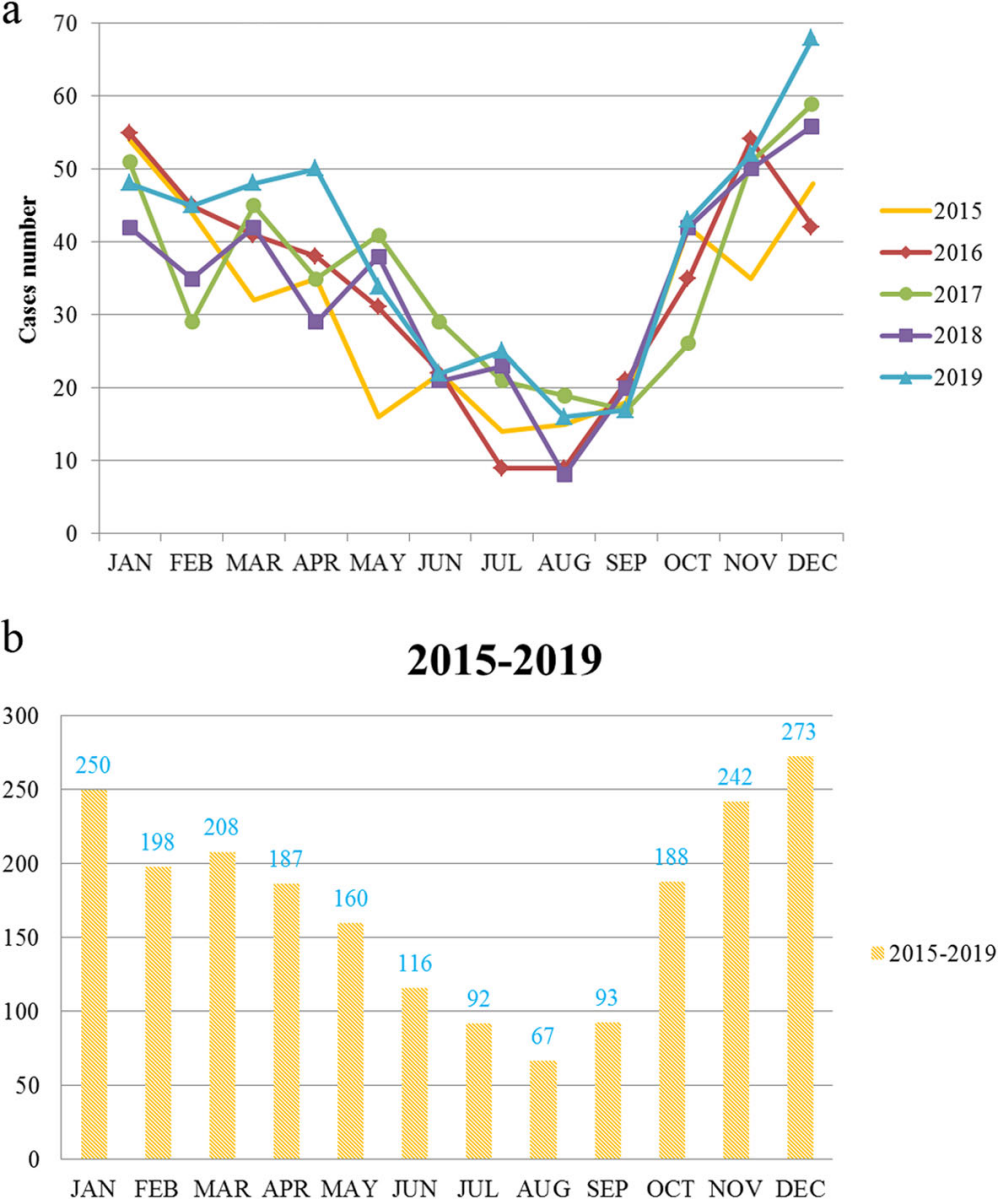
**a** Monthly distribution of 2074 children with HSP from 2015 to 2019. **b** Monthly distribution of 2074 children with HSP from 2015 to 2019

A total of 2074 children suffered from HSP, younger than 17 years old, including 1217 (58.7%) boys and 857 (41.3%) girls from January 2015 to December 2019 (male: female = 1.4:1). The average age was 8.3 ± 3.0 years old and median age was 8 years old (95% CI 8.1–8.4). Interquartile range (IQR) for the onset age fell in the interval between 6 and 10 years old. The annual incidence of HSP was 9.5–10.3 per 100,000, which was calculated according to the demographic data from Anhui Health and Family Planning Commission. The monthly distribution of HSP children is presented in Fig. 1. The highest onset of HSP appeared in January, November or December throughout the observational period; on the contrary, the lowest onset of HSP appeared in July, August or September. In the present study, HSP occurred more commonly in spring and winter than in summer.

### Clinical manifestations

The major clinical manifestations of HSP are shown in Table 1. Since *cutaneous purpura* is the necessary element in the diagnosis of HSP, all patients in the present study had skin lesions. *Arthritis/arthralgia*, the second most prevalent feature of HSP, occurred in 38.4% of patients, resulting in severe pain and even walking restriction. *Abdominal pain* was observed in 35.1% of patients who principally developed gastrointestinal bleeding, intussusceptions, appendicitis or ileus. *Renal involvement* secondary to HSP was diverse and manifested as proteinuria and/or hematuria. In the present study, 621 cases (29.9%) suffered from kidney injury; among them, 161 cases (7.8%) had proteinuria, 153 cases (7.4%) had hematuria and 307 cases (14.8%) had hematuria and proteinuria simultaneously. Besides, *other clinical manifestations* including cardiac damage (3.9%) and liver dysfunction (1.0%) were also found in the present study.

**Table 1:** Clinical manifestations and possible triggers in HSP on admission

	Number of cases (%)
**Clinical manifestations**	
Purpura	2074 (100.0%)
Arthritis/arthralgia	796 (38.4%)
Abdominal pain	729 (35.1%)
Renal involvement	621 (29.9%)
Proteinuria	161 (7.8%)
Hematuria	153 (7.4%)
Hematuria plus proteinuria	307 (14.8%)
**Possible triggers**	
**Infection**	**1030 (49.7%)**
Infectious sites	
Respiratory tract infection	926 (44.6%)
Gastrointestinal infection	103 (5.0%)
Urinary tract infection	20 (1.0%)
Infectious agents	
Streptococcal infection	393 (18.9%)
HP infection	87 (4.2%)
MP infection	84 (4.1%)
Parainfluenza infection	10 (0.5%)
TB infection	4 (0.2%)
RSV infection	3 (0.1%)
Toxoplasma gondii infection	2 (0.1%)
**Allergy**	**289 (13.9%)**
Food allergy	247 (11.9%)
Drug allergy	23 (1.1%)
House dust mite allergy	14 (0.7%)
Grass pollen allergy	5 (0.2%)
**Injury**	**20 (1.0%)**
**Surgery**	**14 (0.7%)**
**Tick bite**	**12 (0.6%)**
**Vaccination**	**8 (0.4%)**
**Unknown**	**886 (42.7%)**

### Possible triggers

The possible triggers of HSP are displayed in Table 1. On admission, series of potential infections were identified in 49.7% of 2074 HSP children. Based on the laboratory results, there were 393 cases (18.9%) with streptococcal infection, 87 cases (4.2%) with HP infection, 84 cases (4.1%) with MP infection, 10 cases (0.5%) with parainfluenza infection, 4 cases (0.2%) with TB infection, 3 cases (0.1%) with respiratory syncytial virus (RSV) infection and 2 cases (0.1%) with toxoplasma gondii infection. Besides infection, 289 cases (13.9%) had allergy, 20 cases (1.0%) had injury, 14 cases (0.7%) had surgery, 12 cases (0.6%) had tick bite, 8 cases (0.4%) had vaccination. However, triggers could not be identified in 886 HSP children (42.7%) yet.

### Association of streptococcal infection with clinical manifestations

**Fig. 2:**
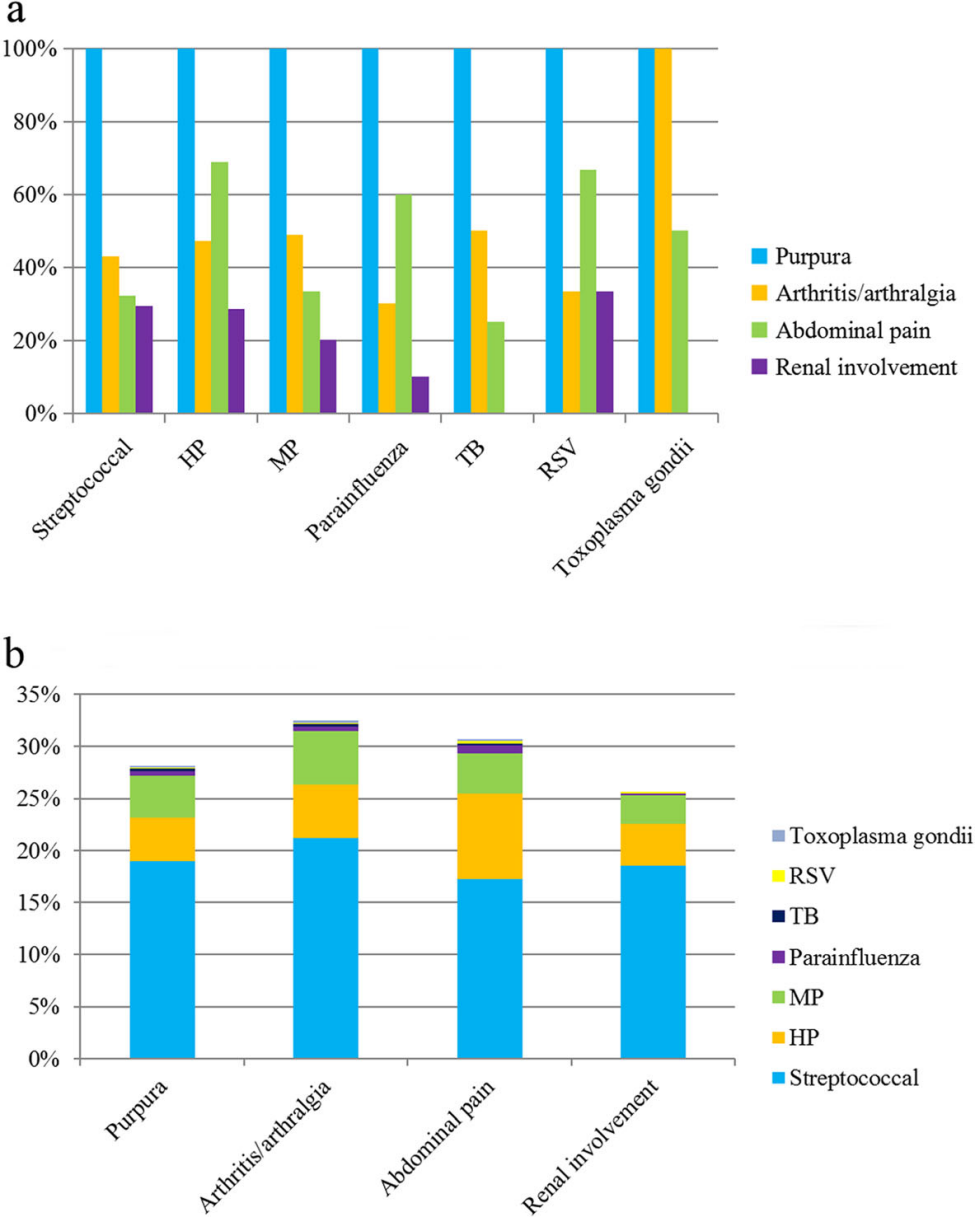
**a** Association of infectious agents with clinical manifestations. **b** Association of infectious agents with clinical manifestations

The association of infectious agents with clinical manifestations in HSP patients is shown in Fig. 2. No significant variations were found between the infectious agents and clinical manifestations (*χ*^*2*^ = 27.0, *p* > 0.05). Among the 393 cases with streptococcal infection, 43.0% of them had arthritis/arthralgia, 32.1% had abdominal pain and 29.3% had renal involvement (Fig. 2a). On the other hand, streptococcus was the most prevalent infectious agent in HSP children regardless of clinical phenotype, and detected in 21.2% of cases with arthritis/arthralgia (796 cases), 18.9% of cases with purpura (2074 cases), 18.5% of cases with renal involvement (621 cases), and 17.3% of cases with abdominal pain (729 cases) (Fig. 2b).

### Influence of streptococcal infection to the therapeutic response of HSP

The remission of main symptoms among the streptococcal infectious group, the other infectious group and the non-infectious group are presented in Table 2. Significant differences in the remission of purpura and renal involvement were noted among the above three groups. More specifically, the remission rate of purpura and renal involvement were significantly higher in the streptococcal infectious group than that in the non-infectious group (*p* < 0.05), but significantly lower than that in the other infectious group (*p* < 0.05). The duration time of purpura and renal involvement were significantly higher in the non-infectious group than that in the streptococcal infectious group and the other infectious group (*p* < 0.05), whereas no significant differences were observed between the streptococcal infectious group and the other infectious group (*p* > 0.05). In addition, there were no significant differences among the three groups with respect to the remission of arthritis/arthralgia and abdominal pain (*p* > 0.05).

**Table 2:** Influence of streptococcal infection to the therapeutic response of HSP

	Streptococcal infection (***n*** = 393)			The other infections (***n*** = 637)			Non-infection (***n*** = 1044)		
	**Total cases**	**Relieved cases (%)**	**Duration time (day)**	**Total cases**	**Relieved cases (%)**	**Duration time (day)**	**Total cases**	**Relieved cases (%)**	**Duration time (day)**
**Clinical manifestations**									
Purpura	393	303 (77.1%)	5.9 ± 4.0	637	525 (82.4%)	5.6 ± 3.8	1044	726 (69.5%)	5.0 ± 3.7
Arthritis/arthralgia	169	169 (100%)	2.5 ± 1.9	293	293 (100%)	2.7 ± 2.2	334	332 (99.4%)	2.5 ± 1.6
Abdominal pain	126	126 (100%)	2.7 ± 2.4	246	246 (100%)	3.2 ± 2.7	357	353 (98.9%)	2.8 ± 2.8
Renal involvement	115	56 (48.7%)	6.4 ± 4.8	149	83 (55.7%)	7.2 ± 5.8	357	154 (43.1%)	5.0 ± 4.9
	**Total cases**		**Frequency (times)**	**Total cases**		**Frequency (times)**	**Total cases**		**Frequency (times)**
**Relapse/recurrence**	76		1.9 ± 1.8	130		1.6 ± 1.4	335		2.6 ± 2.0

The duration of follow-up ranged from 2 weeks to 143 weeks, with a median of 3 weeks. The relapse/recurrence among the three groups is demonstrated in Table 2. Out of 2074 HSP children, 541 (26.1%) were hospitalized more than one time from January 2015 to December 2019. The number of relapse/recurrence ranged from 1 to 10 with a mean of 2.2 ± 1.9 during the observational period. The relapse/recurrence occurred over a time span ranged from 2 weeks to 140 weeks, with a median of 3 weeks (IQR 2–3.5) after initial resolution of symptoms. In 541 cases with relapse/recurrence, there were 76 cases (3.7%) with streptococcal infection, 130 cases (6.3%) with the other infections and 335 cases (16.2%) with non-infection respectively. In the other 1533 cases (73.9%) without relapse/recurrence, there were 317 cases (15.3%) with streptococcal infection, 507 cases (24.4%) with the other infections and 709 cases (34.2%) with non-infection respectively. Significant difference in the relapse/recurrence was found among the three groups. In more detail, the relapse/recurrence rate was significantly lower in the streptococcal infectious group than that in the other infectious group and the non-infectious group (*p* < 0.05). Furthermore, the frequency of relapse/recurrence was significantly higher in the non-infectious group than that in the streptococcal infectious group and the other infectious group (*p* < 0.05), whereas no significant difference was observed between the streptococcal infectious group and the other infectious group (*p* > 0.05).

### Association of streptococcal infection with renal pathology

**Fig. 3:**
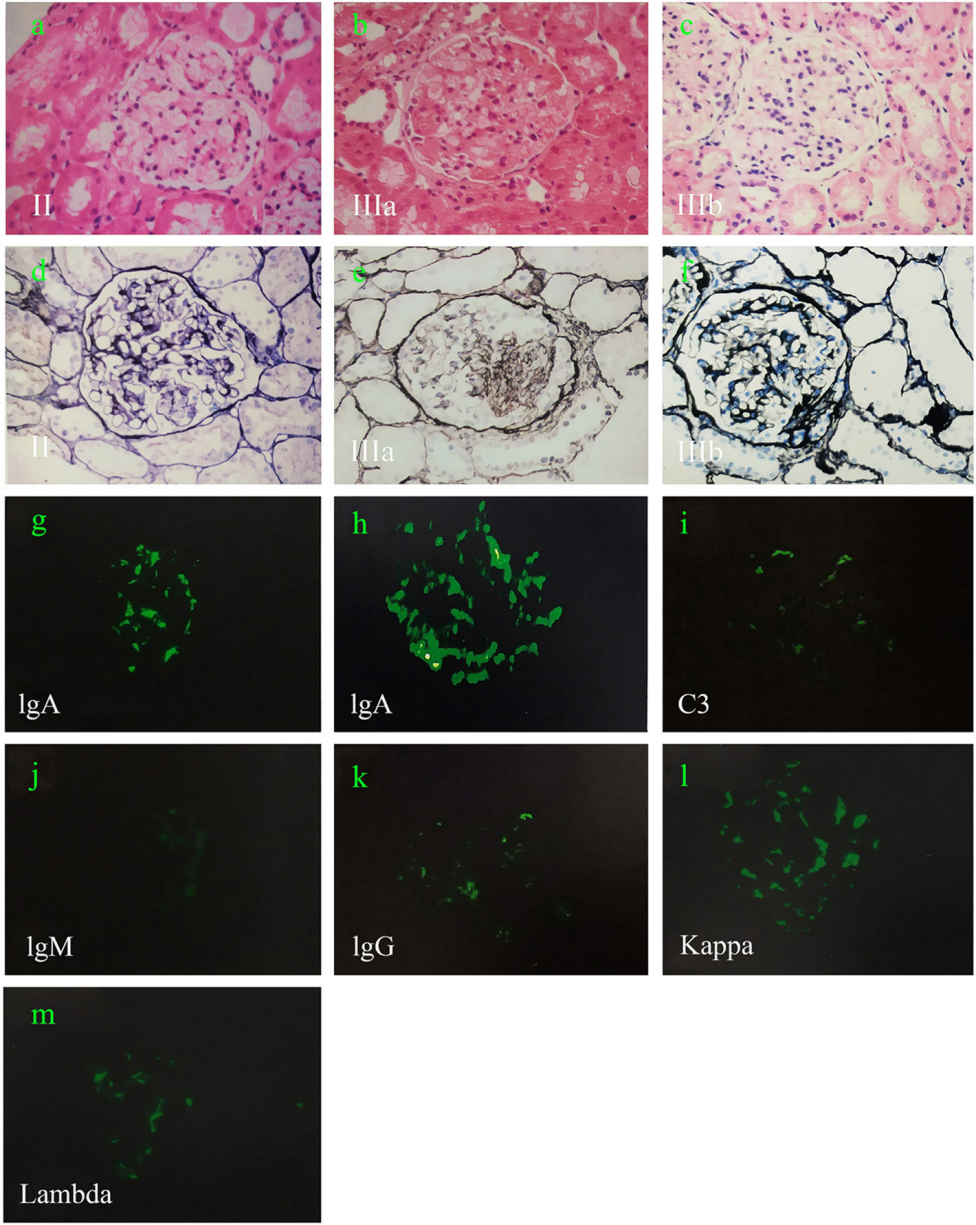
Representative renal pathological images obtained from 42 HSPN cases. **a**-**f** Class II, IIIa and IIIb, light microscopy, glomerulus (**a**-**c**: hematoxylin and eosin stain, **d**-**f** Schiff-methenamine silver stain, original magnification × 400). **g**-**m** Immune complex deposits (**g** 2+ staining for IgA, **h** 3+ staining for IgA, **i** 3+ staining for C3, **j** 1+ staining for IgM, **k** 2+ staining for IgG, **l** 3+ staining for kappa, **m** 3+ staining for lambda, original magnification × 400)

Renal pathological data and representative images are shown in Table 3 and Fig. 3. In the present study, 42 cases with HSPN were subjected to renal biopsy, including 29 boys and 13 girls with the mean age of 10.1 ± 2.7 years old. The biopsy findings according to the ISKDC were as follows: class II: 40.5%; IIIa: 23.8%; IIIb: 35.7%. By immunofluorescence, the hallmark of HSPN consisted of diffuse mesangial deposition of IgA (100%), and to some extent, co-deposition of kappa (78.6%), lambda (76.2%), C3 (59.5%), IgM (42.9%) and IgG (28.6%). However, no significant differences in the ISKDC classification and immune complex deposits existed among the three groups (*p* > 0.05).

**Table 3:** Association of streptococcal infection with renal pathology

	Streptococcal infection (***n*** = 8)	The other infections (***n*** = 7)	Non-infection (***n*** = 27)
**ISKDC**	**Cases**	**Cases**	**Cases**
**II**	4	2	11
**IIIa**	1	1	8
**IIIb**	3	4	8
**C3**	4	6	15
**IgA**	8	7	27
**IgM**	2	4	12
**IgG**	1	2	9
**Kappa**	6	4	23
**Lambda**	6	4	22

## Discussion

In the present study, we recruited 2074 Chinese children with HSP in the recent 5 years. The annual incidence of HSP was 9.5–10.3 per 100,000 based on our local demographic data, with a peak age between 6 and 10 years old. HSP occurrence had a dramatic seasonal variation and more cases occurred in spring and winter than in summer, coinciding with respiratory tract infection [25–27]. The major findings included that: (1) streptococcus served as the most frequent infectious trigger in our subjects; (2) besides purpura, arthritis/arthralgia was the most common clinical manifestation in these cases having streptococcal infection; (3) clearance of streptococcal infection may significantly decreased the relapse/recurrence of HSP; (4) however, renal pathological damage was not aggravated by streptococcal infection.

The etiology of HSP remains unknown, but several triggers have been documented to participate in its pathogenesis [28]. In the present study, streptococcus was the most prevalent infectious trigger. The detection of ASO titer was conducted in both the acute and convalescent phase in a same patient. As we all know, ASO titer generally rises 1 week following infection, peaks at 3 to 5 weeks, and thereafter, declines to the pre-infection levels at around 8 months. However, Anti-DNase B (ADB) titer peaks at 6 to 8 weeks, begins to fall at 12 weeks, and returns to normal levels by 12 months [29]. Therefore, the time option of increased ASO titer seems earlier in the setting of streptococcal infection and more coincident to the average duration of purpura (4.63 ± 2.07 days) in HSP patients, as compared with ADB [11]. Accumulative evidence has indicated that a significant rise in ASO titer from acute phase to convalescent phase serves as a definitive proof of the preceding streptococcal infection [20, 29, 30]. The longest duration of increased ASO titer is almost 8 months after the initial infection of streptococcus. On this background, no enough evidence aids us to judge whether a streptococcal infection 8 months prior to HSP can be identified as a high-level trigger. The local detection of streptococcal transcripts in skin or renal biopsies may be a promising approach to overcome this confusion. Up to now, a total of 9 relevant studies from different administrative areas in China have been reported (Table 4). The incidence of streptococcal infection in HSP children was 7.5% in Taipei [31], 8.7% in Changzhi [32], 9.7% in Lanzhou [33], 12.8% in Dalian [34], 19.7% in Dongying [35], 29.1% in Taiyuan [36], 34.5% in Soochow [37], 47.5% in Jinan [38] and 48.2% in Laiwu [39], respectively; obviously, our finding fell in the above range, similar to the data from Dongying [35]. The advantages of our study are mainly large sample size and observations of long duration. However, the association between streptococcal infection and HSP is still controversial. Ayoub et al. [40] studied 33 HSP patients, and observed that no significant differences in streptococcal serology existed between HSP patients and their controls.

**Table 4:** Streptococcal infection in Chinese patients with HSP from different administrative areas

Areas	Period (years)	Total cases	Infection	Respiratory tract infection	Cases having ASO test	Elevated ASO	Reference
Taipei	10	261	–	–	53	4 (7.5%)	[31]
Changzhi	3	337	68 (34.9%)	64 (32.8%)	195	17 (8.7%)	[32]
Lanzhou	2	325	186 (57.2%)	119 (36.6%)	196	19 (9.7%)	[33]
Dalian	4	141	–	114 (80.9%)	141	18 (12.8%)	[34]
Dongying	2	71	–	35 (49.3%)	71	14 (19.7%)	[35]
Taiyuan	2	502	262 (68.8%)	232 (60.9%)	381	111 (29.1%)	[36]
Soochow	1	338	–	165 (48.8%)	165	57 (34.5%)	[37]
Jinan	3	120	73 (60.8%)	52 (43.3%)	120	57 (47.5%)	[38]
Laiwu	3	56	–	–	56	27 (48.2%)	[39]
Hefei	5	2074	1030 (49.7%)	926 (44.6%)	2074	393 (18.9%)	Our study

In general, arthritis/arthralgia serves as the most frequent clinical manifestation of HSP besides purpura, followed by abdominal pain and renal involvement respectively [4, 41]. In a 5-year retrospective study conducted by Mao et al. [2] from 2009 to 2013, purpura, arthritis/arthralgia, abdominal pain and renal involvement occurred in 100, 57.8, 49.9 and 49.8% of Chinese patients with HSP, respectively, which was consistent with our finding. However, to the best of our knowledge, few studies about the association of streptococcal infection with clinical manifestations in HSP patients have been reported to date. On this background, the present study focused on this issue, and found that 43.0, 32.1 and 29.3% of cases manifested arthritis/arthralgia, abdominal pain and renal involvement in 393 HSP patients triggered by streptococcal infection, respectively; thus, streptococcal infection seems no influence on the clinical manifestations of HSP. Multisystem involvement in HSP patients triggered by streptococcal infection may be attributed to antigenicity of different bacterial components of streptococcus through mediating host immune dysfunction. More specifically, the hyaluronic acid capsule stimulates antibody cross-reactivity against joint tissues [42]; streptococcal M-protein specific T-cells are transmigrated into heart tissue through its interaction with the heart endothelium, which results in the endothelial cell activation and inflammatory process aiming to cardiovascular system [43]; in addition, streptococcal M protein and carbohydrate antigen (N-acetyl-beta-D-glucosamine) share antigenic epitopes with human cardiac myosin and laminin on heart valves [14].

Although HSP is considered as a self-limited disease, 2.7 to 51.7% of patients experience relapse/recurrence with a mean follow-up of 22 years [44]. In addition, Bui et al. [45] reported 2 patients with Wegener granulomatosis masqueraded as HSP at time of initial presentation. However, in the present study, no patient met the diagnostic criteria of other vasculitis during the follow-up. Numerous observational studies have consistently showed that HSP patients having a frequent relapse/recurrence are more likely to develop nephritis and significant proteinuria [15, 46]. Lei et al. [5] reviewed the clinical data of 1020 HSP patients in Taiwan, China from 1997 to 2012, and discovered that 16.1% of HSP patients experienced 1 relapse and 8.5% of HSP patients experienced 2 relapses. Rigante et al. [47] followed 74 Italian patients with HSP for 5 years, and found that 9 of them presented at least 1 relapse, in which almost half of all cases had renal involvement. In the past 2 decades, several epidemiological surveys have been devoted to finding some risk factors possibly related to relapse in HSP, including older age (> 10 years), persistent purpura (> 1 month), long-term steroid treatment (> 10 days), allergic rhinitis, gastrointestinal involvement and HSPN [5, 46–49]. In the present study, 26.1% of HSP patients relapsed or recurred more than 1 time within a 5-year observational period, and the relapse/recurrence rate in streptococcal infectious group was subjected to a 0.4-fold decrease as compared with the non-infectious group; thus, a history of streptococcal infection appears a protective factor for relapse/recurrence of HSP. In addition, the relapse/recurrence rate in the other infectious group was subjected to a 0.36-fold decrease as compared with the non-infectious group. Clearance of the other infections may also significantly decrease the relapse/recurrence of HSP. However, a retrospective study encompassing 206 Korean HSP patients by Shin et al. [46] indicated that the relapse/recurrence rate in patients with streptococcal infection was not significantly lower than their counterparts; and the association between streptococcal infection and relapse/recurrence of HSP is still controversial.

Renal involvement is a major contributor to the long-term prognosis of HSP, and usually presents transitory microscopic hematuria and/or low-grade proteinuria in the first 3 months after HSP diagnosis [50]. A retrospective analysis of 107 Chinese HSP patients from 1991 to 2005 performed by Nong et al. [51] showed that 28.0% of them had renal involvement, in which 1.9% had isolated proteinuria, 18.7% had isolated hematuria and 7.5% had hematuria combined with proteinuria. Zhang et al. [52] studied 95 biopsy-proven patients with HSPN, and observed that 42.1% of patients were classified as class IIIb, 24.2% of patients with class IIIa, 17.9% of patients with class IIb, 10.5% of patients with class IV, 3.2% of patients with class IIa and 2.1% of patients with class V; and moreover, the most characteristic immunofluorescence finding of HSPN consisted of diffuse mesangial deposits of IgA (97.9%), followed by fibrogen (91.6%), IgG (73.7%), IgM (68.4%), C3 (62.1%), C1q (33.7%) and C4 (12.6%). In the present study, 42 patients with HSPN were subjected to renal biopsy. Patients were treated with cyclophosphamide when the biopsy-proven HSPN nephritis histopathology was greater than class II and the informed consent was obtained from parents simultaneously. Twenty cases were treated with cyclophosphamide, and 16 of them (80.0%) got remission during the observational period, almost 2 folds higher than those who without cyclophosphamide treatment. To date, no biological agent was certificated by the Food and Drug Administration to be prescribed to pediatric HSPN. In this situation, we have no application experience of biological agent to our patients. Several predictive systems have been established to assess the risk of HSPN. Based on the data from 13 studies encompassing 2398 HSP patients, a meta-analysis by Chan et al. [8] indicated that some risk factors were predictive of HSPN, including relapse (OR = 4.7), persistent purpura (OR = 4.0), severe bowel angina (OR = 3.4), decreased C3 (OR = 3.1), age > 10 years (OR = 3.1), platelets > 500 × 10^9^/L (OR = 3.0), WBC > 15 × 10^9^/L (OR = 2.4), elevated ASO (OR = 2.2), abdominal pain (OR = 1.9), gastrointestinal bleeding (OR = 1.9), male gender (OR = 1.4) and older age (OR = 0.9). On this basis, streptococcal infection is recognized as one of HSPN risk factors. Nephritis-associated plasmin receptor triggered by streptococcus has been positively identified to have a diffuse and global distribution in mesangium and may play a pathogenic role in patients with HSPN [53]. However, the present study identified no significant differences in urine tests, ISKDC classification and immune complex deposits among the streptococcal infectious group, the other infectious group and the non-infectious group.

In addition, there are still 3 limitations in the present study: first, patients from a single center may cause selection bias and outcome reporting bias; second, based on the available hospital discharge data alone, almost 20% of patients may be ignored in our clinical trial; third, given the rapid onset of disease and the large number of patients, it was almost impossible to test for streptococcal infection by blood-agar culture for each patient. Therefore, these limitations will be overcome in our subsequent study.

## Conclusions

Streptococcal infection is the most frequent trigger for childhood HSP and does not aggravate renal pathological damage; the possible elimination of streptococcal infection helps relieve the relapse/recurrence of HSP.

## Data Availability

The datasets generated and/or analysed during current study are available from the corresponding author on reasonable request.

## Abbreviations

HSP: Henoch-Schönlein purpura
IgAV: Immunoglobulin A vasculitis
HSPN: Henoch-Schönlein purpura nephritis
C3: Component 3
ASO: Antistreptolysin O
HP: Helicobacter pylori
MP-Ab: Mycoplasma antibodies
TB-Ab: Tubercle bacillus antibody
TORCH: Toxoplasma gondii, others, rubella virus, cytomegalo virus, herpes virus
EBV: Epstein-Barr virus
CK: Creatine kinase
AST: Aspartate aminotransferase
ALT: Alanine aminotransferase
ISKDC: International Study for Kidneys Disease in Children
IQR: Interquartile range
RSV: Respiratory syncytial virus
ADB: Anti-DNase B
